# Initial evidence of presence of papillary muscle diffuse fibrosis in patients with cardiomyopathy

**DOI:** 10.1186/1532-429X-17-S1-P284

**Published:** 2015-02-03

**Authors:** Shingo Kato, Sébastien Roujol, Jihye Jang, Tamer A Basha, Sophie Berg, Kraig V Kissinger, Goddu Beth, Warren J Manning, Reza Nezafat

**Affiliations:** Beth Israel Deaconess Medical Center, Boston, MA USA; Yokohama City University Hospital, Yokohama, Japan

## Background

The left ventricular (LV) papillary muscles (PM) are important structure for mitral valve complex. Previous studies by echocardiography demonstrated that PM function was impaired in patients with hypertrophic cardiomyopathy (HCM) and dilated cardiomyopathy (DCM). Native T_1_ mapping has emerged as a noninvasive magnetic resonance (MR) imaging method to assess LV diffuse myocardial fibrosis without using contrast agent. The aims of this study were to evaluate feasibility and reproducibility of PM native T_1_ measurement, and to compare PM T_1_ times between cardiomyopathy patients and controls subjects.

## Methods

Seventeen cardiomyopathy patients (11 HCM; 6 DCM, age: 57 ± 15 years) and 16 control subjects (age: 50 ± 15 years) were enrolled. Native T_1_ mapping images were acquired with MOLLI (Messroghli DR et al. MRM 2004) sequence in 3 short-axis planes (basal, mid and apical slices) using an ECG-triggered single-shot acquisition with a balanced SSFP readout (TR, 3.1; TE, 1.5; FA, 35°; FOV, 360 × 337 mm^2^; acquisition matrix, 188 × 135; voxel size, 1.9 × 2.5 mm^2^; slice thickness, 8 mm). All data were corrected for motion (Roujol S et al. MRM 2014). Measurement of PM T_1_ was performed both in anterior PM and posterior PM in all subjects. Intra-observer and inter-observer reproducibility was assessed by 2 readers in 20 PMs from 10 control subjects. Repeatability coefficients were calculated as 1.96 times the standard deviation of the differences on the Bland-Altman plots.

## Results

Intra- and inter- observer reproducibility of PM native T_1_ measurement was high. Repeatability coefficient of 10.2 msec, intraclass correlation coefficient (ICC) of 0.99 (95% confidence interval (CI): 0.99 -1.00, p<0.05) and coefficient of variation (CV) of 0.36% for intra-observer reproducibility. Repeatability coefficient of 15.4 msec, ICC of 0.99 (95% CI: 0.99 -1.00, p<0.05), CV of 0.63% for inter-observer reproducibility. Comparing cardiomyopathy patients and control subjects, significant difference was found both in anterior PM T_1_ value (1102 ± 43 msec vs 1102 ± 90 msec, p=0.003) and posterior PM T1 value (1100 ± 69 msec vs 1010 ± 69 msec, p=0.002).

## Conclusions

Measurement of PM native T_1_ is feasible and reproducible. This MR approach successfully detected abnormal PM T1 value in cardiomyopathy patients, and could provide new insight into possible pathophysiological mechanism of PM dysfunction in various cardiovascular diseases.

## Funding

Shingo Kato, MD receives scholarship from Banyu Life Science Foundation International.

**Figure 1:**
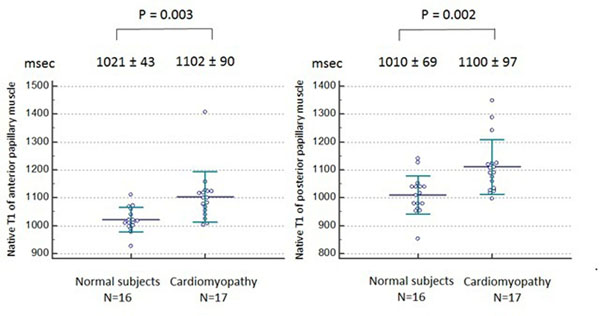
**Comparison of papillary muscle T1 between cardiomyopathy patients and control subjects.** Significant difference was found in native T1 value both in anterior papillary muscle and posterior papillary muscle between control subjects and cardiomyopathy patients.

